# Premature Dentition and Supernumerary Teeth

**Published:** 1848-01

**Authors:** J. L. Levison


### ARTICLE X.

### *Premature Dentition and Supernumerary Teeth.*

To the Editor of The London Lancet.

SIR,—Some time since I was asked by a medical friend to see a female infant, born with two central incisores of the lower jaw, particularly as she suffered from great local irritation. When we called, the child was not very ill, but very fretful; the mouth was hot; there was an extreme secretion and flow of saliva, but the parents objected to the teeth being then extracted. At the end of six weeks from my first visit, the symptoms became more alarming. There was a violent attack of diarrhœa, with indications of a general affection of the mucous membranes of the alimentary canal. The child looked thin, pale, and somewhat emaciated, from constant pain. Having explained that the disturbance was clearly attributable to the continued irritation of the teeth, (which were extraneous bodies,) the mother consented that they should be removed. It was almost marvellous, the improvement in a few days. All the above mentioned symptoms left her, and she acquired plumpness, color, and strength.

Some writers on premature dentition assert that such teeth are generally very defective in their composition; that the dentine is but imperfectly formed; that there is not a vestige of enamel; and that it is very doubtful whether there is any substance analogous to the crusta petrosa. These statements are generally correct; but in the one mentioned above, the teeth are not only in a normal state, so far as the dentine is concerned, but the whole of the crowns and the fangs are covered with a beautiful, highly polished enamel, very dense, and of a brilliant white color. The fangs of each were perfectly formed, being of the usual length of such, and similar to those which make their appearance at the ordinary period.

It would appear that the vital energy of the vascular nervous pulps of these kind of teeth is so vigorous, as to cause their formation and rapid development: but, as if from this very circumstance, the vitality of the nervo-sanguineous pulp, with its secreting powers, seems to be by such means exhausted; for it is rarely that such premature teeth are completely formed. The two I have just described are the most perfect of any in my possession, yet they are not tapered to a point. In each tooth the pulp could be seen (by means of a microscope) somewhat contracted, and somewhat tapering, but still within the bony cavity; so that from being thus within the bony envelope, they became, to all intents and purposes, merely extraneous bodies.

My infantile patient was healthy and well formed, with black hair on her head at her birth, in a rather larger quantity than is usually the case. Her parents were healthy, and about thirty years old. They had married early, and had many other children, whose teeth were not developed before the usual period.

Some of our dramatists have attributed evil tendencies to children who are born with "teeth," but my little patient was subsequently amiable and tractable. Nor could I find any solution from any preternatural growth of other organs, having particularly examined the osseous structure, but without detecting any marked difference when compared with other infants of a similar age.

*Supernumerary Teeth.*—I would also call your attention to another curious fact in the dental system—the formation of supernumerary teeth, which can only be regarded as a connate variety, similar to the occasional development of six fingers and six toes.

Supernumerary teeth are found of various shapes: some conical, some pear-shaped, some resembling other teeth, with some modification of structure. They are usually formed between the central incisores, or between the lateral incisores, and in some instances in the palatine bones. There is one great peculiarity—they have usually a greater thickness of enamel than any other teeth in the same mouth. In some instances they are connected with the posterior or the lateral surfaces of incisor teeth, and appear as offshoots of one of the tooth pulps. I recollect one instance where the supernumerary tooth connected the two central incisores. In the numerous instances in which I have extracted them, I do not remember more than one exception in which they have been found before the development of the second or permanent teeth. Lately, I removed one from a young lady of a delicate form, with a nervous temperament, the shape of which resembled a lateral incisor, very thickly coated with enamel, particularly on its posterior surface, in which that substance was in thick masses, and ribbed. There could not be any difficulty as to its being a supernumerary tooth, as there were two large central incisores, (one forced in an oblique direction,) with two beautifully formed lateral incisores on each side; the cuspidati, bicuspides, and molares, being of the usual number and character.

I am, sir, yours, respectfully,

J. L. LEVISON.

*Quackery*, is the tail end of progression, and bears the same relation to civilization that the goose does to the giblets—sensible men swallow the former, whilst the latter is the dish of ignorant menials and satraps.—Gideon Harvey.

